# Use of Medicare Benefit Scheme mental health services in young people who experienced self-harm and/or suicidal behaviours: Data from the Young Minds Matter survey

**DOI:** 10.1177/10398562231163415

**Published:** 2023-03-19

**Authors:** Kate M. Chitty, Michael Gifford Sawyer, Gregory Carter, David Lawrence

**Affiliations:** Biomedical Informatics and Digital Health, Faculty of Medicine and Health, 4334 The University of Sydney, Sydney, NSW, Australia; Professor of Child and Adolescent Psychiatry, School of Paediatrics and Child Health, 1066 University of Adelaide, Stirling, WA, Australia; Conjoint Professor in Psychiatry in the Faculty of Health Sciences, 5982 University of Newcastle, Callaghan, NSW, Australia; Professor of Mental Health, School of Population Health, 1649 Curtin University, Perth, WA, Australia

**Keywords:** healthcare, health services, suicidal behaviour, mental health, general practice

## Abstract

**Objectives:**

To examine healthcare utilisation patterns in a sample of young people with self-reported experiences of self-harm and/or suicidal behaviours.

**Methods:**

A national survey examining mental health in a nationally representative sample of young Australians aged 12–17 years, linked to routinely collected healthcare and dispensing data. For respondents that self-reported experience of self-harm, suicidal ideation, suicidal plan and/or suicide attempt, we assessed attendance at a Medicare Benefits Scheme (MBS) subsidised MH service or non-MH general practitioner (GP) attendance at three time periods: 1) ever, 2) in the 12 months prior to completing the survey and 3) after completing the survey until 31 Dec 2015. We also assessed correlates associated with attendance and non-attendance at a MH service.

**Results:**

The study included 311 young people. MH services were attended in the 12 months before the survey by 38.3% with attempted suicide, 28.7% with a suicidal plan, 28.9% with suicidal ideation and 29.4% with self-harm. MH treatment administered by a GP was the most common MH service (25%); followed treatment by psychologist (15%) and psychiatrist (5%). Attendance at a MH service was observed highest alongside more severe self-reported depression.

**Conclusions:**

Potential underutilisation of MBS MH services by young people with self-harm and/or suicidal behaviours.

Self-harm and suicidal behaviours are common in young people and are associated with significant levels of distress. The Young Minds Matter survey of Australian young people estimated that 10.9% of 12–17 year olds, or 186,000 young people, reported they had self-harmed at some point in their life and 8.0% reported they had self-harmed in the 12 months prior to the survey.^1^

Suicide prevention is a significant public health priority and the country’s approach is set out in The Fifth National Mental Health and Suicide Prevention Plan.^2^ The Plan is underpinned by eight priority areas to achieve improved mental and physical health and effective suicide prevention and mental health (MH) service delivery is a strong underlying component. The Plan proposes that Australians with severe mental illness (estimated at approximately 3% of the population) should be provided multidisciplinary clinical care as well as appropriate pharmacotherapy, inpatient services and psychosocial support services.

Many individual and systemic barriers to help-seeking exist, especially in young people. Individual barriers include lack of faith that available supports can help, lack of knowledge of available supports and associated costs.^3^ Major systemic barriers include lack of available services, limited government funding, lack of knowledge/training in mental health/suicide prevention in the workforce and fragmented health policies.^2^ The Plan recognises that many people in Australia with severe and complex mental illness do not receive the supports they need; however, there are no robust estimates of healthcare access by young people that experience suicidal behaviours.

The aim of this study was to use data from the Young Minds Matter survey, linked to administrative health claims captured by Australia’s Medicare Benefits Scheme (MBS) and Pharmaceutical Benefits Scheme (PBS), to describe patterns of service use before and after the survey.

Specifically, the study aims to

1. Determine the proportion of young people with self-reported self-harm and/or suicidal behaviours in the prior 12 months who attended an MBS-funded MH service or non-MH consultation with a general practitioner (GP), which services were attended and how many times the service was attended in the prior 12 months.
2. Describe characteristics of young people who had ever experienced self-harm and/or suicidal behaviours who had and had not attended an MBS-funded MH service.

## Methods

### Data

This study used data from Young Minds Matter: The second Australian Child and Adolescent Survey of Mental Health and Wellbeing. The methodology for the study has been described elsewhere.^4^ Briefly, a national household survey was conducted in 2013–2014, with 6,310 randomly selected Australian families. Face-to-face interviews were conducted in parents of 4–17 year olds and a self-report questionnaire completed by children and adolescents aged 11–17 years. The current study uses data for children and adolescents aged 12–17 years.

Survey data was linked to individual records from the MBS and PBS from the child’s birth until 31 December 2015. Under the MBS all Australian citizens and residents receive subsidised healthcare for out-of-hospital medical services. The PBS reimburses community pharmacies and private hospitals for PBS-listed medicines for all Australian citizens and residents and the subsidy is passed on to the consumer.^5^

### Outcomes

The four categories of behaviour assessed included: self-harm (without suicidal intent), suicidal ideation, suicidal plan and suicide attempt. These categories were reported for lifetime and for 12 months prior to the survey. See Supplementary Material for specific questionnaire items.

Any GP consultation that did not carry a MH code was considered a non-MH GP attendance. MH contacts were classified by any MBS record for a psychologist or psychiatrist consultation, MH treatment delivered by a GP, a GP consultation with a corresponding prescription date for a psychoactive medicine (in the PBS record) or MH treatment delivered by other health workers. Outcomes were MH attendance ever (Y/N), MH contact in the year prior to survey (Y/N) and MH attendance in the time since the survey until 31 December 2015 (Y/N).

### Independent variables

Sociodemographic factors were measured using the parent interview and included: age, sex, annual household income and index of relative socio-economic advantage and disadvantage quintile based on home address.

Parents and young people were independently administered the Diagnostic Interview Schedule for Children-IV (DISC-IV).^6^ Parents were administered DISC-IV modules for seven disorders (major depressive disorder [MDD], four anxiety disorders, attention deficit hyperactivity disorder and conduct disorder) and young people were administered the youth MDD module to determine diagnosis and severity.^7^

### Data analysis

We used descriptive statistics to determine the proportion of 12–17 year olds who self-reported experience of self-harm, suicidal ideation, suicidal plan or suicidal attempt in the 12 months prior to the survey who received MBS-funded MH services in their lifetime, 12 months prior to the survey and after the survey (until 31 December 2015). We reported the types of services attended in the 12 months prior as well as the number of attendances per service.

### Ethics

The research protocol for the study was approved by the Australian Government Department of Health Human Research Ethics Committee (HREC) and UWA Human Research Ethics Committee HREC. Approval to access MBS and PBS data with consent was provided by the Australian Government Department of HREC and the External Request Evaluation Committee of the Australian Government Department of Human Services.

## Results

### Sample characteristics

**Figure 1.**
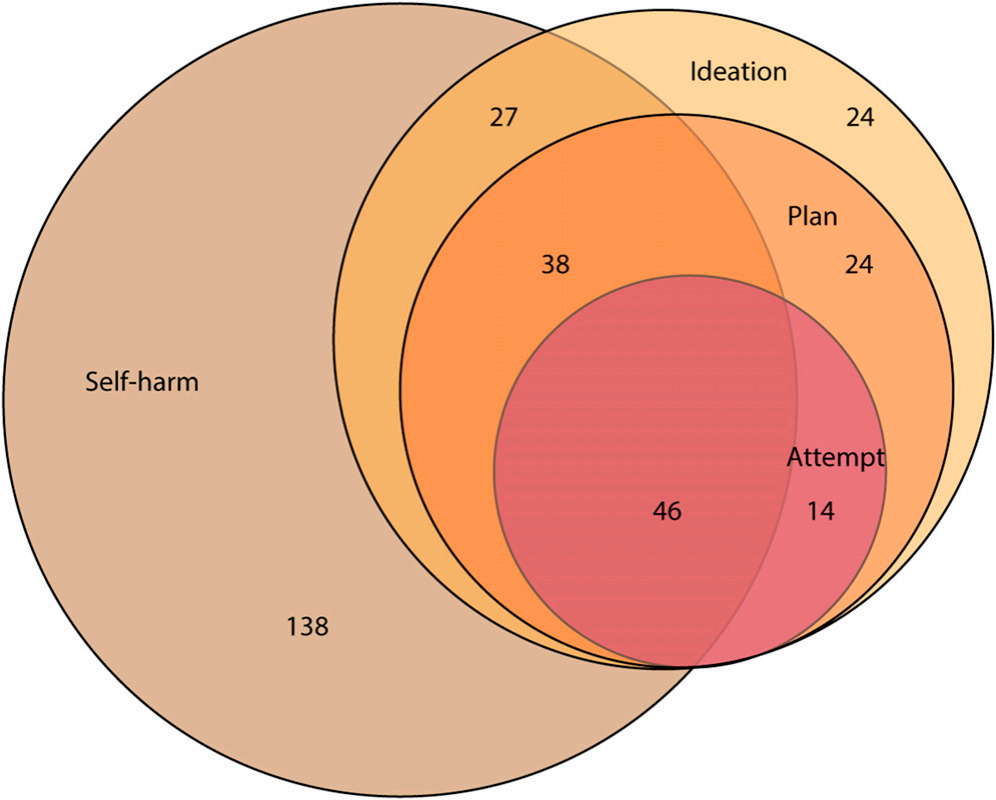
Venn diagram depicting the overlap between respondents who reported ever experiencing self-harm or the various suicidal behaviours. Because of the way the questions were sequenced, everyone who attempted was assumed to have a plan, and everyone who had a plan was assumed to have suicidal ideation.

The sample comprised of 311 young people (14.1% of total number of 12–17 year olds from the original Young Minds Matter survey with linked data available); their characteristics are shown in Table 1. Figure 1 shows a Venn diagram depicting the overlap between respondents who reported ever experiencing self-harm or the various suicidal behaviours. Of those who had ever experienced self-harm or suicidal behaviours, 266 had experienced these in the 12 months prior to the survey, specifically 194 reported self-harm, 173 reported ideation, 122 reported a plan and 60 reported an attempt.

**Table 1.** Characteristics associated with different MBS-funded mental health service patterns by 12–17 year olds who have ever self-harmed or considered suicide

		*n*	Period of MBS or PBS funded mental health service use			
			Never	Ever	In the 12 months prior to the survey	Since the survey^1,2^
Sex	Male	112	52 (46.4)	60 (53.6)	26 (23.2)	36 (40.5)
	Female	199	75 (37.7)	124 (62.3)	69 (34.7)	100 (57.1)
Age group (years)	12–14	76	45 (59.2)	31 (40.8)	13 (17.1)	11 (37.9)
	15–17	235	82 (34.9)	153 (65.1)	82 (26.4)	125 (53.2)
Household income (AUD)	<52,000	82	27 (32.9)	55 (67.1)	23 (28.1)	39 (60.0)
	52,000–129, 999	138	61 (44.2)	77 (55.8)	45 (32.6)	60 (49.2)
	≥130,000	83	36 (43.4)	47 (56.6)	23 (27.7)	34 (48.6)
Socio-economic status (quintile)	Lowest	54	23 (42.6)	31 (57.4)	14 (25.9)	21 (52.5)
	Second	58	20 (34.5)	38 (65.5)	16 (27.6)	28 (56.0)
	Third	62	23 (37.1)	39 (62.9)	23 (37.1)	33 (56.9)
	Forth	61	29 (47.5)	32 (52.5)	14 (23.0)	21 (42.0)
	Highest	76	32 (42.1)	44 (57.9)	28 (36.8)	33 (50.0)
Parent report of any psychiatric disorder in their child^3^	Yes	97	14 (14.4)	83 (85.6)	58 (59.8)	62 (71.2)
	No	214	113 (52.8)	101 (47.2)	37 (17.3)	74 (41.8)
Youth self-report of major depressive disorder^3^	Yes	142	38 (26.7)	104 (73.2)	61 (43.0)	85 (63.0)
	No	169	89 (52.7)	80 (47.3)	34 (20.2)	51 (39.5)
Both parent and youth report of major depressive disorder?	Yes	60	8 (13.3)	52 (86.7)	36 (60.0)	43 (71.7)
	No	251	119 (47.4)	132 (52.6)	59 (23.5)	93 (37.0)
Severity of youth self-reported major depressive disorder^3^	None	169	89 (52.7)	80 (47.3)	34 (20.1)	51 (39.5)
	Mild	28	13 (46.4)	15 (53.6)	5 (17.8)	13 (48.2)
	Moderate	51	16 (31.4)	35 (68.6)	19 (37.3)	26 (54.2)
	Severe	63	9 (14.2)	54 (85.7)	37 (58.7)	46 (76.7)
Total		311	127 (40.8)	184 (59.2)	83 (26.7)	124 (47.0)

^1^Data not available for 12–13 year olds due to consent requirements (figures based on 264 14–17 year olds).

^2^Covers the period from date of survey to 31 December 2015.

^3^As determined via administration of the Diagnostic Interview Schedule for Children-IV (DISC).

### Health service attendance

**Figure 2.**
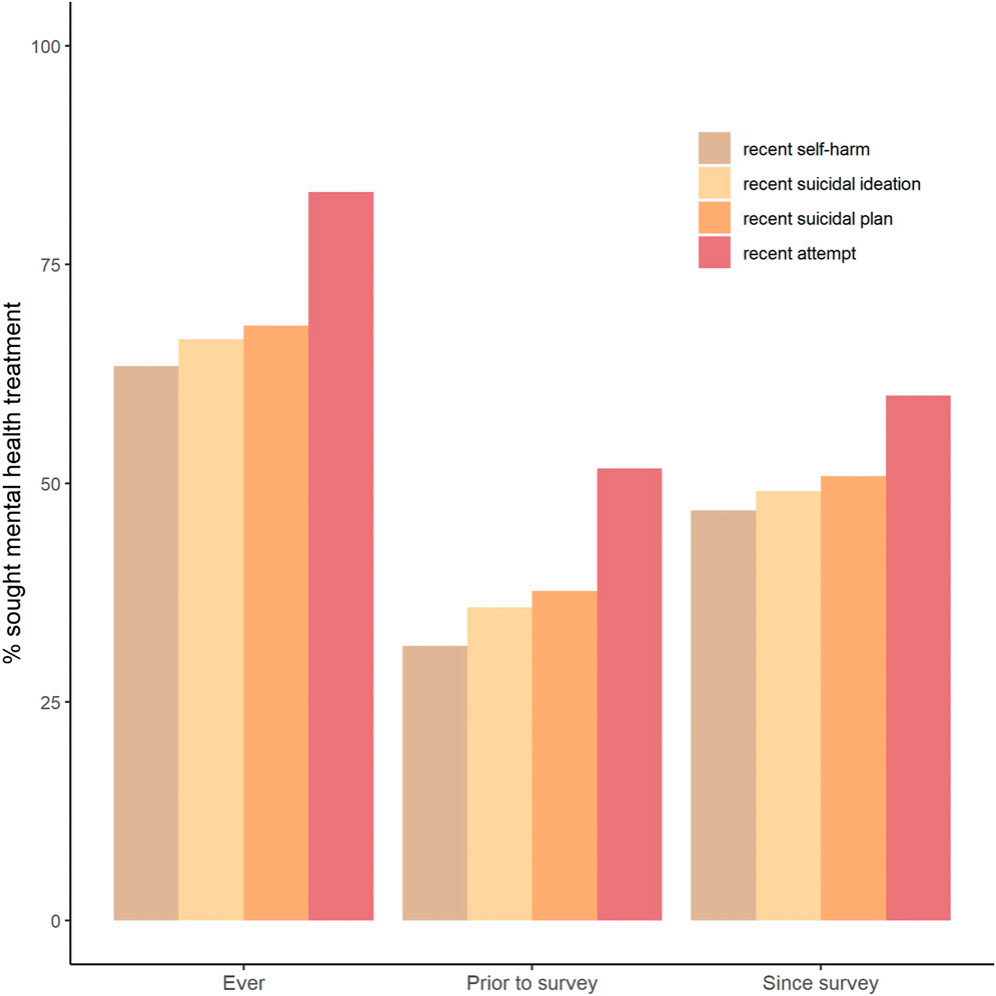
The percentage of 12–17 year olds who displayed self-harm, had a suicide plan, had experienced suicidal ideation and/or attempted suicide in the 12 months prior to the survey that used MBS or PBS funded mental health services ever, in the 12 months prior to the survey and after the survey.

Figure 2 shows the proportion of 12–17 year olds who recently displayed self-harm, had a suicide plan and had experienced suicidal ideation or attempted suicide that attended MBS-funded MH services ever, in the 12 months prior to the survey and after the survey.

**Figure 3.**
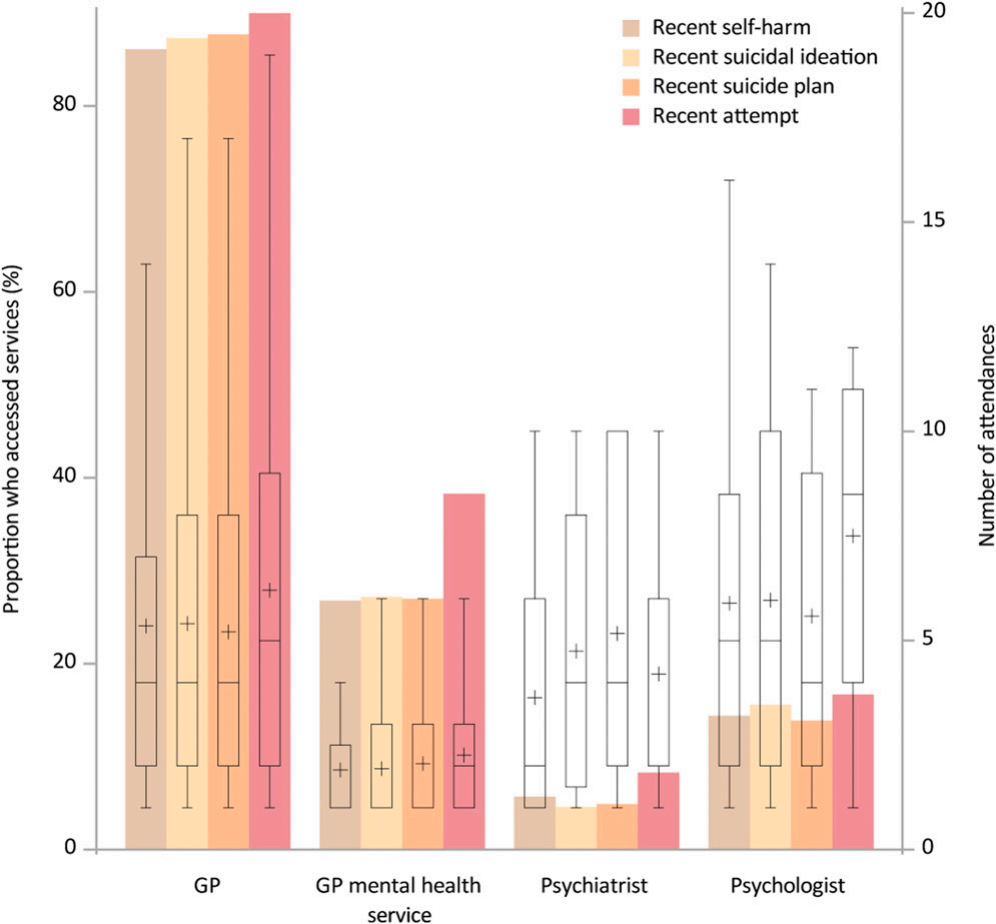
Bars correspond to the primary *y*-axis and show the percentage of 12–17 year olds who displayed self-harm, had a suicide plan, experienced suicidal ideation or attempted suicide in the 12 months prior to the survey and the different types of services they accessed. Box and whisker plots corresponding to the secondary *y*-axis overlay the bars and represent the distribution in terms of number of attendances in the 12 months prior to the survey per service of those who accessed that service. Crosses represent the mean.

Figure 3 displays the proportion of 12–17 year olds who had recently displayed self-harm, had a suicide plan, had experienced suicidal ideation or attempted suicide and the different types of services they attended in the 12 months prior to the survey, and the number of times that service was attended over the timeframe.

Table 1 shows the characteristics associated with different MBS funded MH service attendance patterns of the sample. The medicines dispensed to the sample are shown in Supplementary Material.

## Discussion

This study presents an overview of MBS-subsidised MH services and non-MH GP attendance in young people who self-reported experiencing self-harm and/or suicidal behaviours.

Only 29.4% of young people who self-harmed within 12 months of the survey had accessed MBS-funded MH treatment during the same timeframe, 28.7% of young people with a recent suicide plan and 38.3% of young people with a recent attempt. There is limited data to compare these estimates, as it is the first time to our knowledge that a large MH survey in young people has been linked to administrative data. However, studies with linked healthcare data for suicide decedents have reported similar MH service access in the year before death; around 25–33%.^8–10^ For those attending, the average number of sessions with a psychologist (around 6) or a psychiatrist (3–5) are consistent with guidelines on what might constitute a minimally effective treatment for children or adolescents with mental disorders.^11^

Though marginal, the lower service use observed by those who self-harm or have suicidal ideation compared to those having attempted suicide may reflect that many adolescents and their families wait until a suicide attempt occurs to seek professional help. Likewise, more severe self-reported MDD was seen alongside higher proportion of MH service attendance. Indeed, the First Australian Government’s National Suicide Prevention Taskforce has identified that a system that relies on health services response at the point of crisis is one that misses many opportunities for suicide prevention and that a refocus toward early identification of distress in addition to clinical interventions is necessary.^12^

Over 75% of the respondents who had displayed self-harm or suicidal behaviour in the 12 months prior to the survey had also seen a GP within the same timeframe, and GP MH specific appointments were the most common MH treatment. This highlights the important role of GPs as gatekeepers for self-harm and suicidal behaviours that are driven by MH and non-MH related factors. Self-reported MDD was not identified during the diagnostic interview in around half the young people and any psychiatric disorder was not identified during the diagnostic interview in 69% of their parents. These findings may highlight social determinants of self-harm and suicidal behaviours may be prominent in this age group and alternative approaches for prevention, outside of the MH treatment framework, are necessary. This shift in focus is supported by the recent advice delivered to the Australian Government by the National Suicide Prevention Taskforce.^13^

Like many studies that have looked at sex differences in service use, we find a greater proportion of females accessed services compared to males. Older adolescents had higher rates of service use in all time periods when compared to younger adolescents, which likely represents increasing severity of underlying MH problems over time and, conversely, delay in recognising and responding to MH problems in younger people.

### Limitations and future directions

There are limitations to this study that should be borne in mind. Firstly, the survey did not identify dates the suicidal behaviours took place and hence it is not possible to identify if the service use occurred before or after the suicidal behaviour. Secondly, due to ethical restraints there was no post-survey data linkage for children aged 12 and 13 years and therefore sample numbers are small and not representative. Thirdly, MBS data does not capture all MH services, and therefore this study represents an under-estimate. Finally, administrational data linked on the individual level is sensitive and obtaining such data is prone to long delays and as a result the data presented here may not reflect current estimates of suicidal behaviour and MH service access in Australia. A recent systematic review would suggest that rates of psychiatric service use have declined in young people since the COVID pandemic.^14^ Hence, this study should be considered a pilot study that highlights the urgent need for a substantial and contemporary review of MBS-subsidised mental health services in Australia.

## Conclusions

This study highlights underuse of MBS-subsidised MH MBS-subsidised MH services in young people who self-harmed or experienced suicidal behaviours who do not self-report major depression in the Second Young Minds Matter survey. This highlights the importance of reinforcing alternative avenues for suicide prevention, delivered outside the mental health sector. That said, Australia’s National Mental Health Services Planning Framework,^15^ and the Productivity Commission’s recent report into Mental Health^16^ recognise attempted suicide as a marker of severe distress and recommend all individuals attempting suicide should receive MH care. As noted by the Productivity Commission, lack of service use by suicidal individuals is a consistent issue across the lifespan. Conversely, those who self-reported MDD in the study had high MBS-subsidised mental health service use, indicating the need for and importance of accessibility of these services.

## Supplemental Material

Supplemental Material - Use of Medicare Benefit Scheme mental health services in young people who experienced self-harm and/or suicidal behaviours: Data from the Young Minds Matter survey Click here for additional data file. Supplemental Material for Use of Medicare Benefit Scheme mental health services in young people who experienced self-harm and/or suicidal behaviours: Data from the Young Minds Matter survey by Kate M Chitty, Michael Sawyer, Gregory Carter, and David Lawrence in Australasian Psychiatry
