# Supplementary material for: Use of Medicare Benefit Scheme mental health services in young people who experienced self-harm and/or suicidal behaviours: Data from the Young Minds Matter survey
Source: Australas Psychiatry. 2023 Mar 19;31(3):270–6. doi: 10.1177/10398562231163415 (PMC10251470; doi:10.1177/10398562231163415)
Supplement: Supplemental Material - Use of Medicare Benefit Scheme mental health services in young people who experienced self-harm and/or suicidal behaviours: Data from the Young Minds Matter survey [file sj-pdf-1-apy-10.1177_10398562231163415.pdf]

Supplementary material

Outcome definitions

<u>Self-reported self-harm and suicidal behaviours</u>

Self-harm was measured by the following questions: "Have you ever deliberately done something to yourself to cause harm or injury, without intending to end your own life?"; 'Have you deliberately harmed or injured yourself without intending to end your own life during the past 12 months?' Suicidal ideation was measured by the following question, 'Have you ever felt that life was not worth living?'; 'During the past 12 months, did you ever seriously consider attempting suicide?'. Suicidal plan was measured by the following question, "Have you ever actually attempted suicide?'; 'During the past 12 months, did you make a plan about how you would attempt suicide?' Suicide attempt was measured by the answer to the following question: 'How many times have you ever attempted suicide?' 'Did you attempt suicide during the past 12 months?'.

Supplementary table 1: Psychotropic medicines dispensed to the sample in the 12 months prior to the survey and the 18 months after the survey

Anatomical Therapeutic Classification	Medicine class	Prescriber			Average number of prescriptions
		GP	Specialist	Any	
N05A	Antipsychotics	12	11	13	6.3
N05B	Anxiolytics	5	6	8	2.2
N05C	Hypnotics and sedatives	7	2	8	1.0
N06A	Antidepressants	78	44	83	9.8
N06B	Psychostimulants	10	11	11	8.7
N06C	Psycholeptics and psychoanaleptics	0	0	0	0
N07B	Drugs used in addictive disorders	3	0	3	2.0
